# Supplementary material for: Preclinical Therapeutic Efficacy of RAF/MEK/ERK and IGF1R/AKT/mTOR Inhibition in Neuroblastoma
Source: Cancers (Basel). 2024 Jun 25;16(13):2320. doi: 10.3390/cancers16132320 (PMC11240493; doi:10.3390/cancers16132320)
Supplement: Supplementary file 1 [file cancers-16-02320-s001.zip › cancers-3008576-supplementary-final/Supplemental Figures S1-S2.pdf]

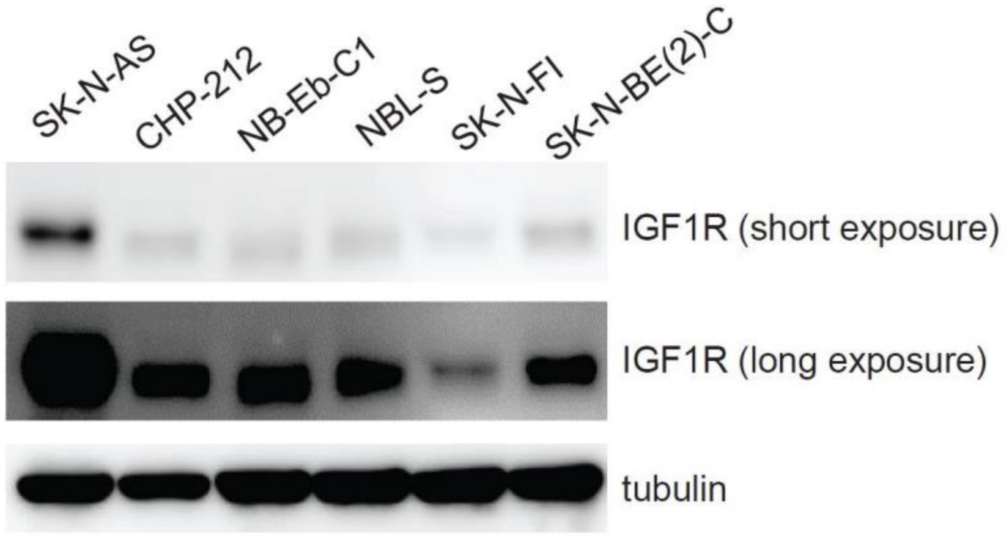

Supplemental Figure S1: Expression of IGF1R in the panel of RAS/MAPK-altered neuroblastoma cell lines as determined by immunoblot. Tubulin is used as a loading control

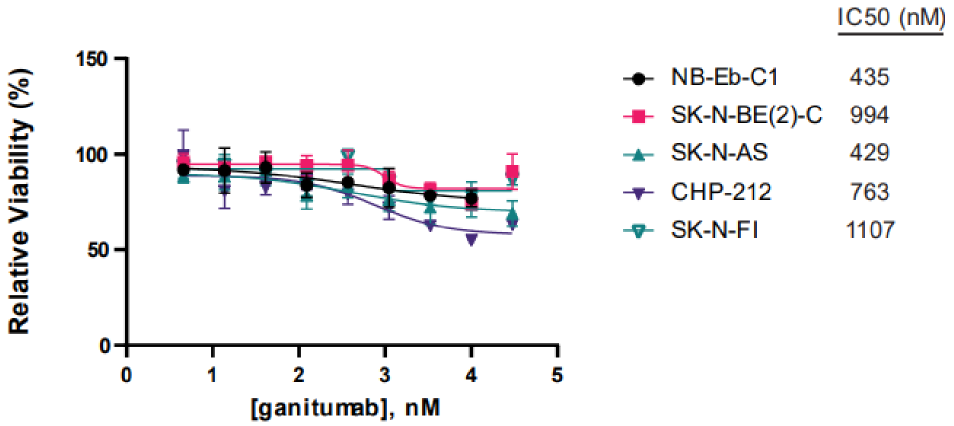

Supplemental Figure S2: Efficacy and potency of ganitumab was determined in RAS/MAPK-altered neuroblastoma cells using CellTiter-Glo signal 72 hours after treatment as a marker of viability. Means of technical triplicates are displayed. Error bars indicate the standard deviation
