# Supplementary material for: Preclinical Therapeutic Efficacy of RAF/MEK/ERK and IGF1R/AKT/mTOR Inhibition in Neuroblastoma
Source: Cancers (Basel). 2024 Jun 25;16(13):2320. doi: 10.3390/cancers16132320 (PMC11240493; doi:10.3390/cancers16132320)

# Figure 1B: NF1

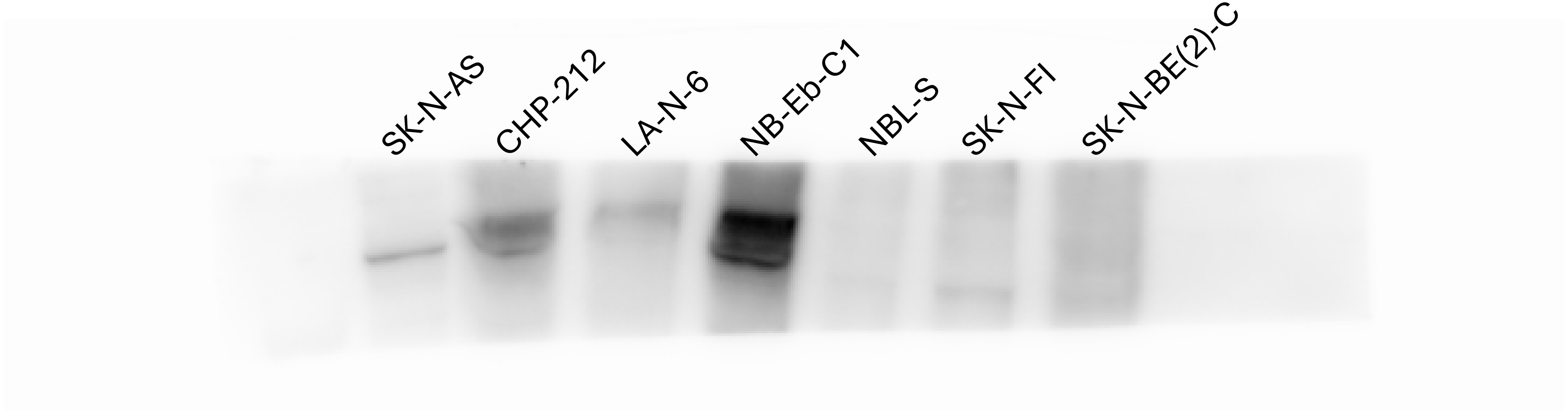

# Figure 1B: vinculin

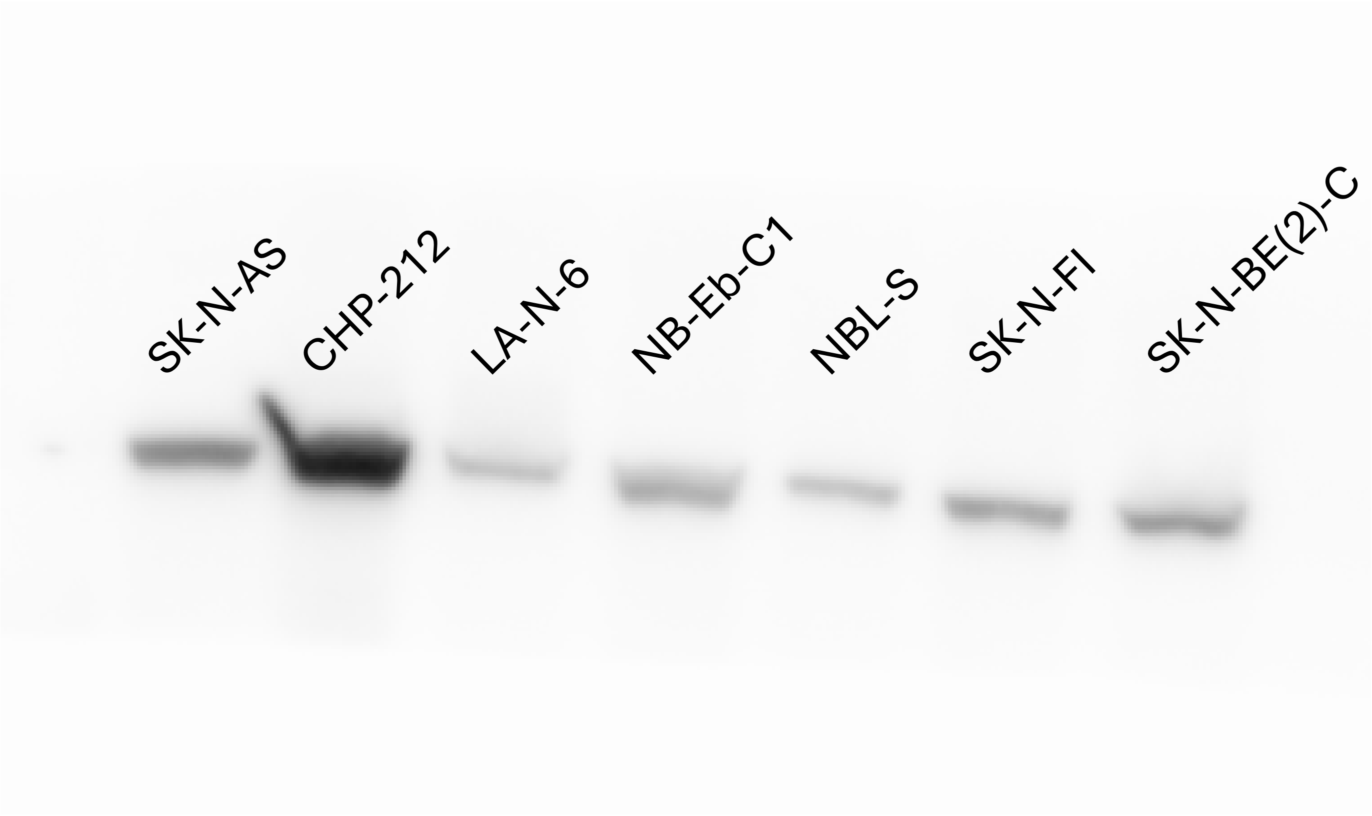

# Figure 2B: SK-N-AS, pERK

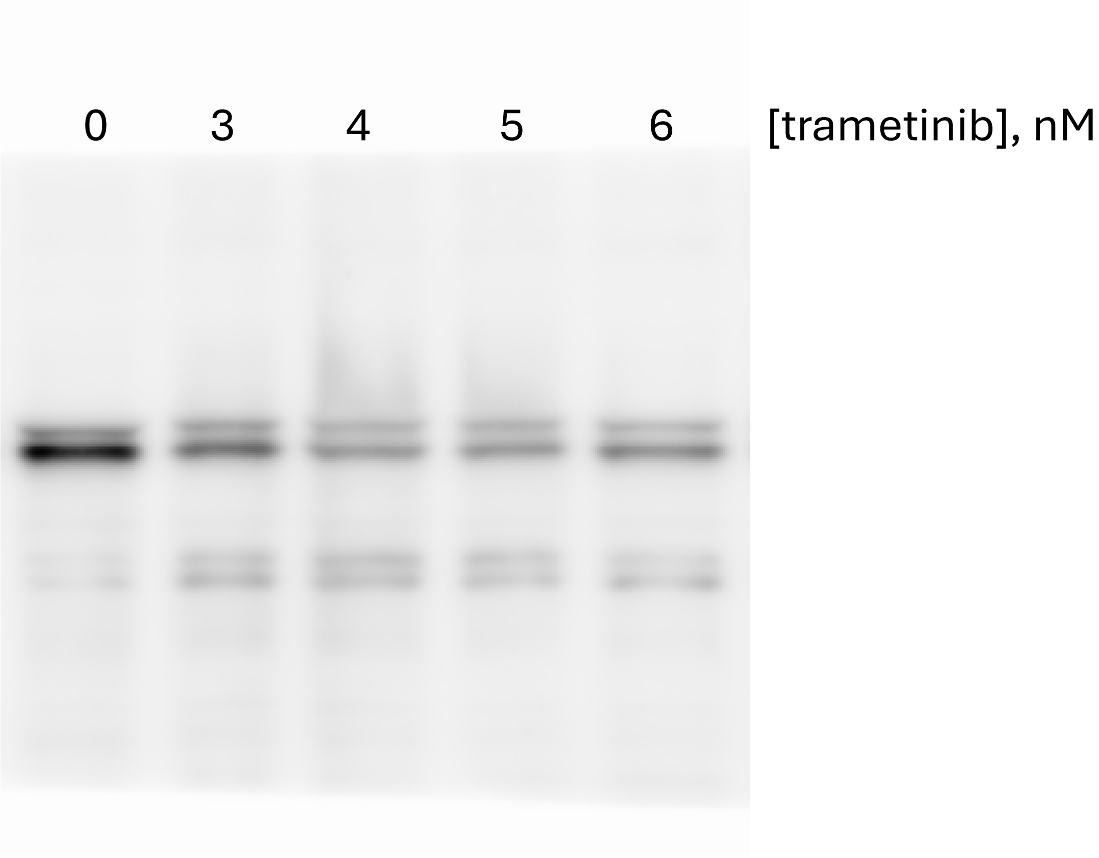

# Figure 2B: SK-N-AS, vinculin

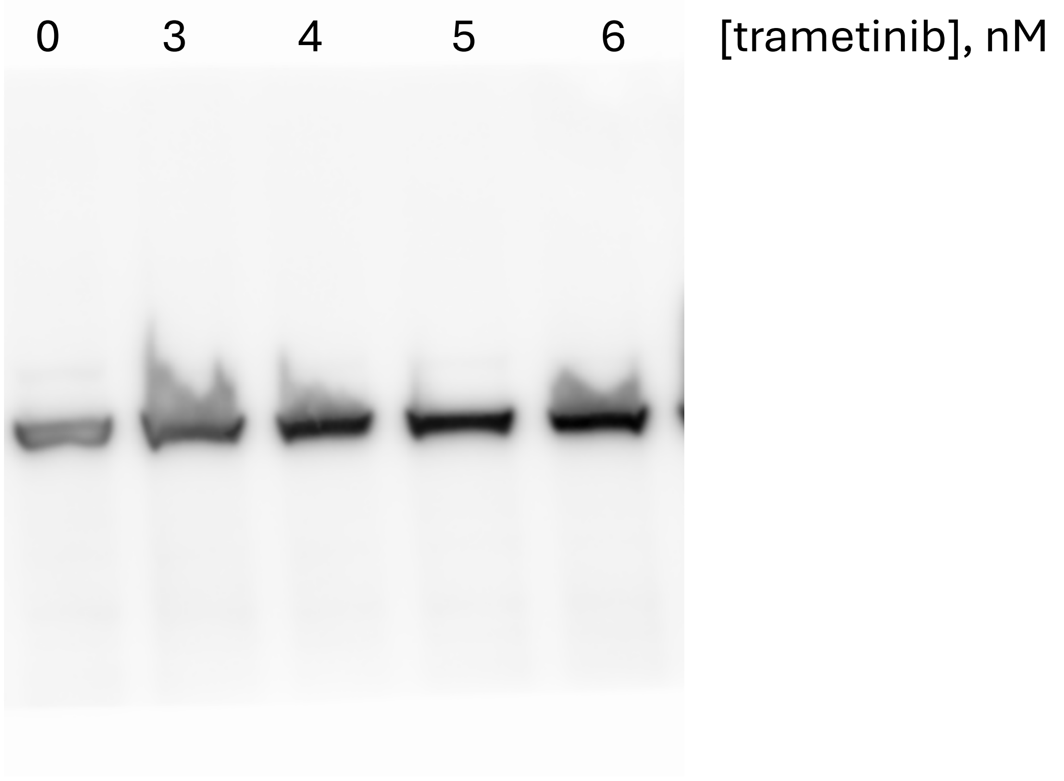

# Figure 2B: CHP-212, pERK

pERK CHP212 fig 2

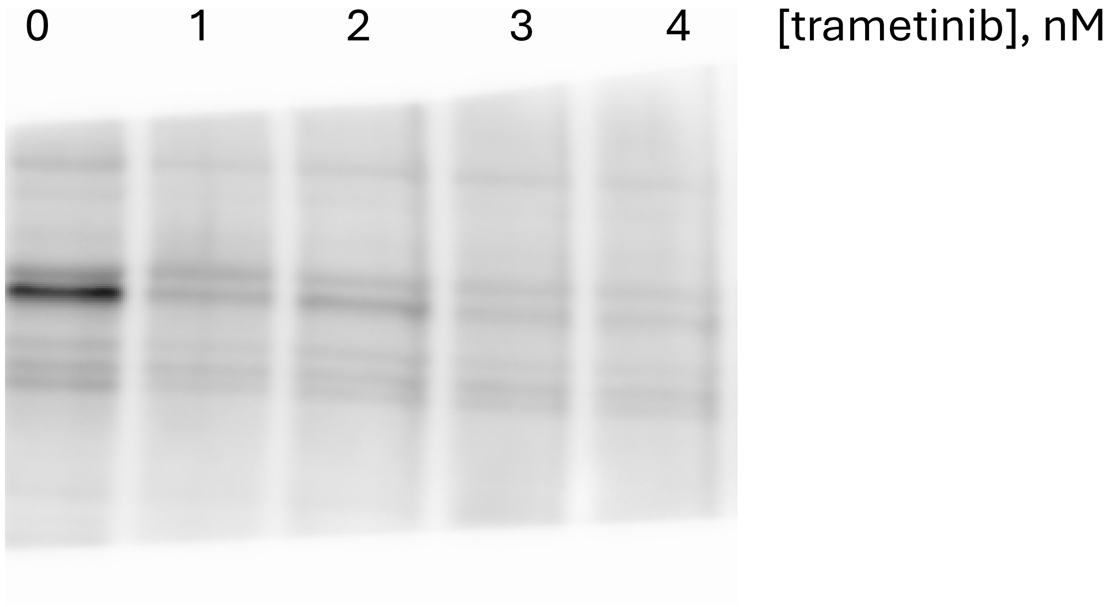

# Figure 2B: CHP-212, vinculin

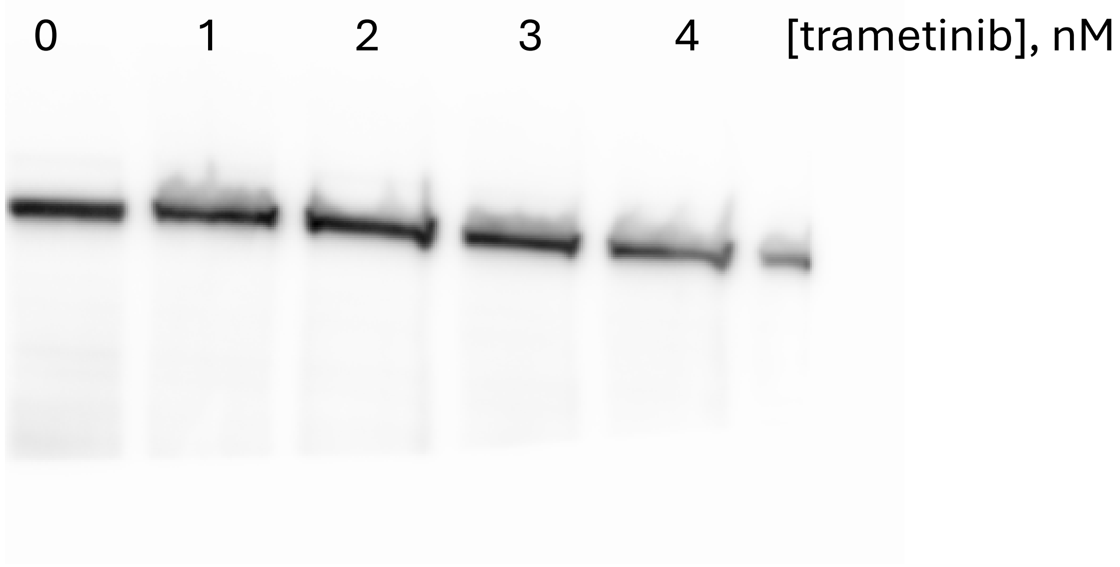

# Figure 2B: NB-Eb-C1, pERK

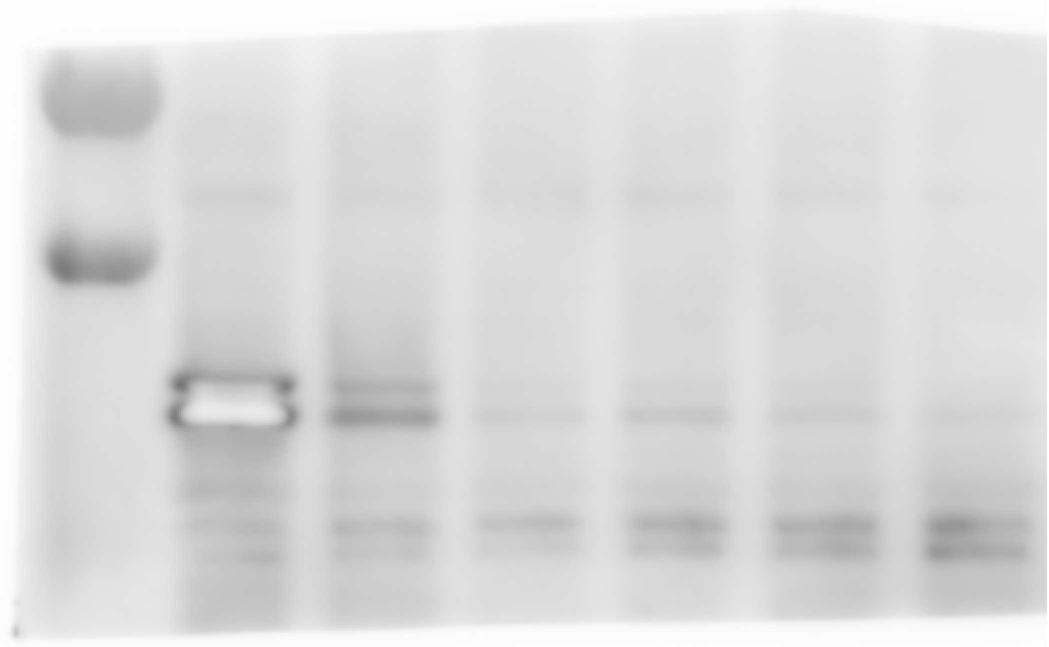

# Figure 2B, NB-Eb-C1, vinculin

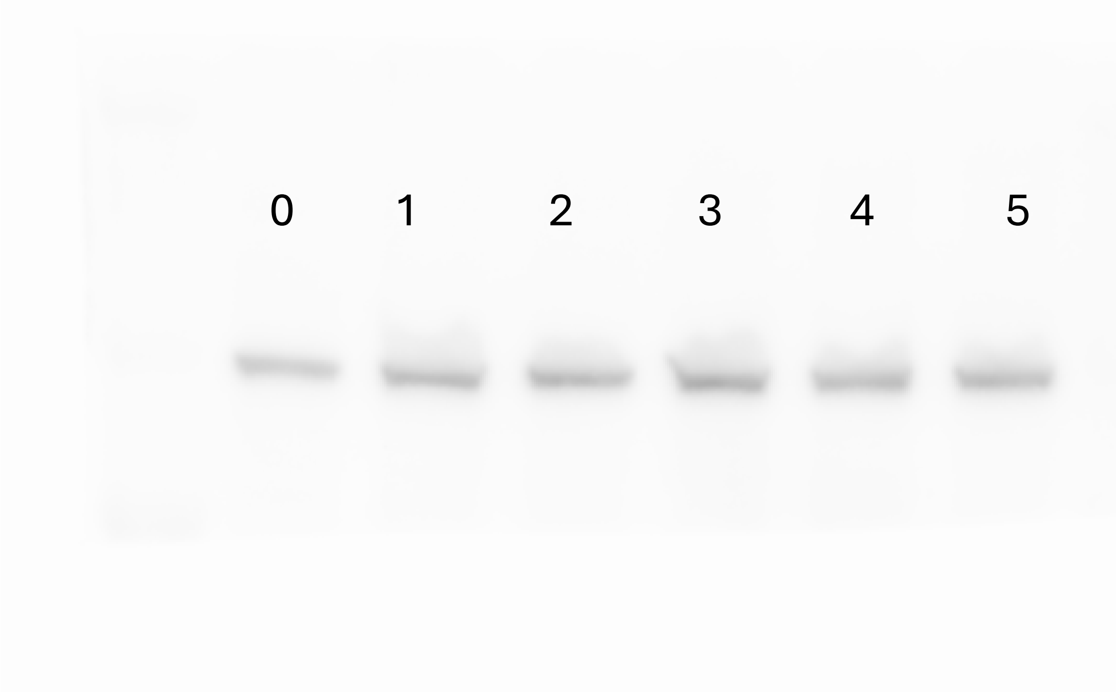

# Figure 2B: LA-N-6, pERK

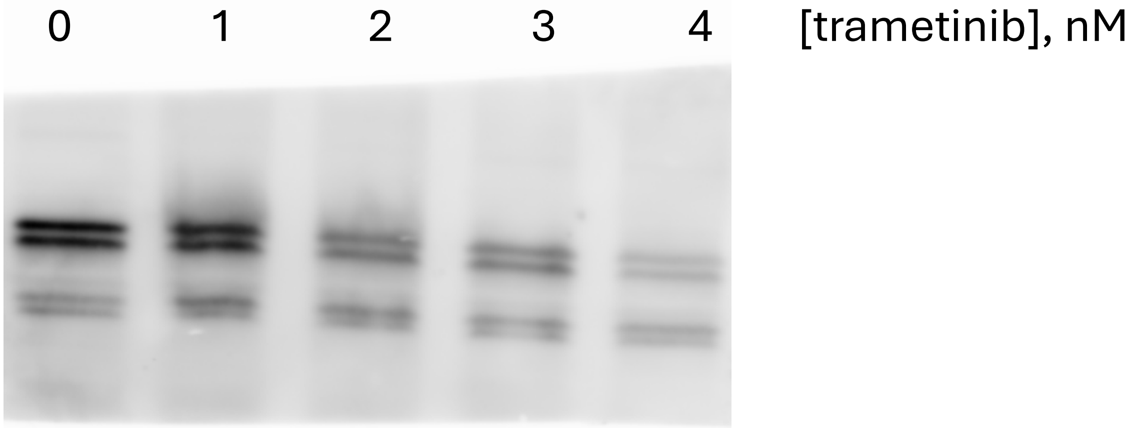

# Figure 2B: LA-N-6, vinculin

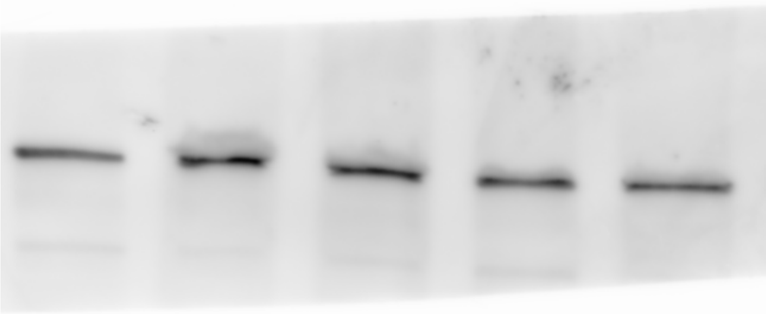

# Figure 2B: SK-N-FI, pERK

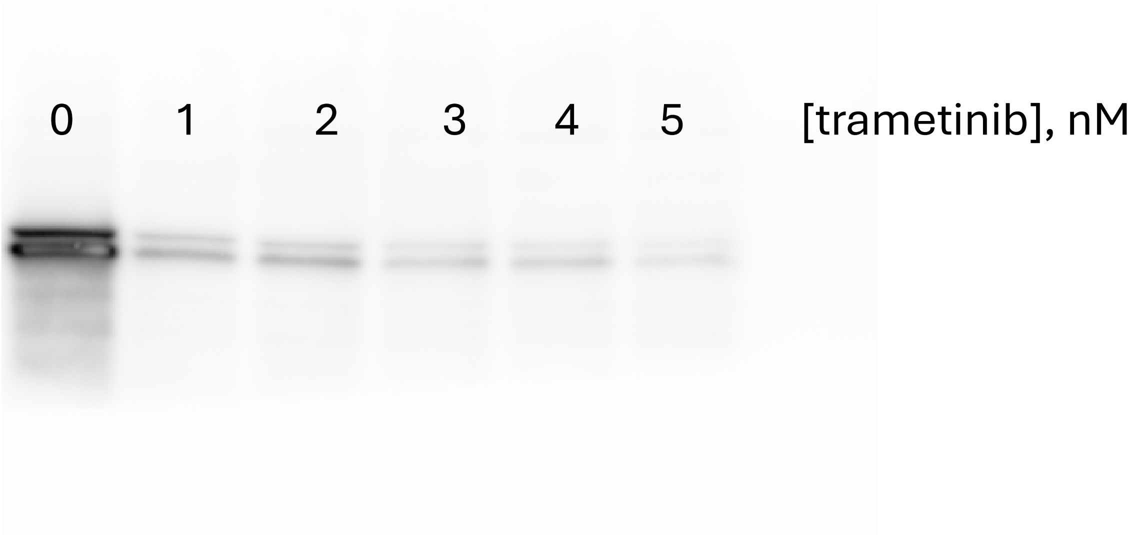

# Figure 2B: SK-N-FI, vinculin

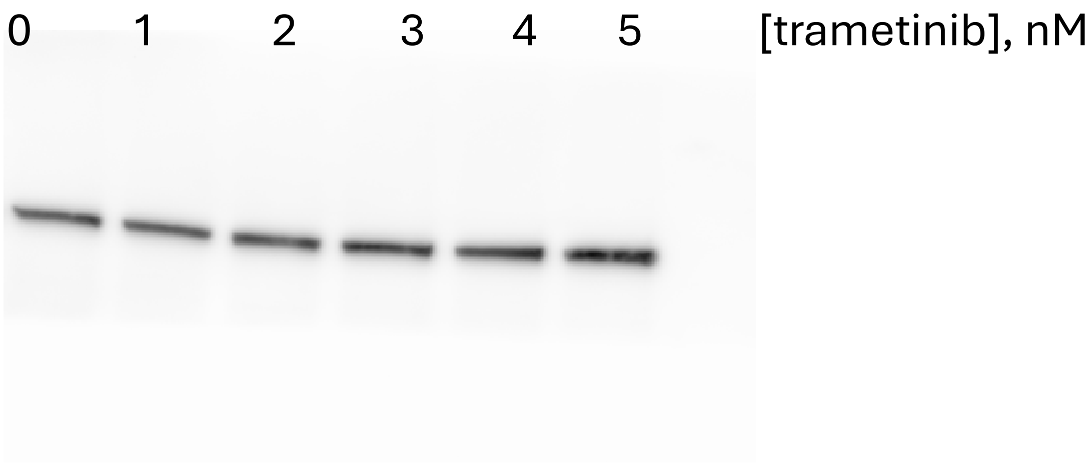

# Figure 2B: NBL-S, pERK

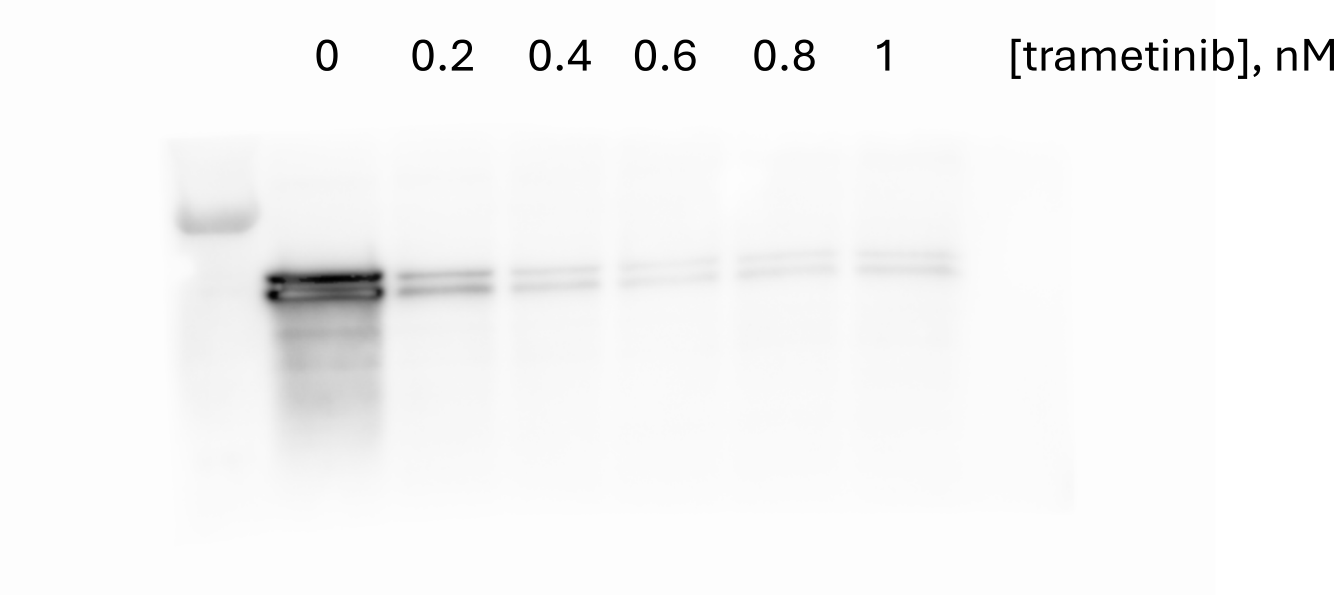

# Figure 2B: NBL-S, vinculin

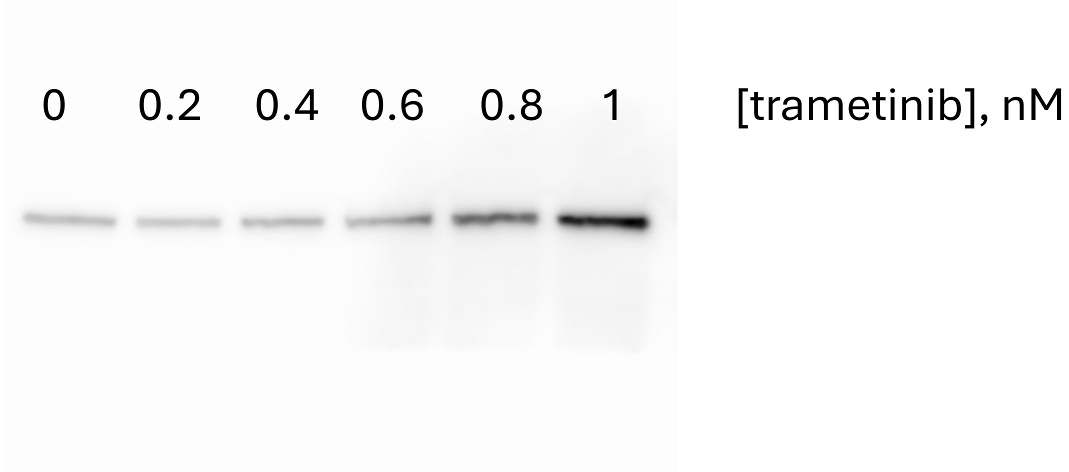

# Figure 2B: SK-N-BE(2)-C, pERK

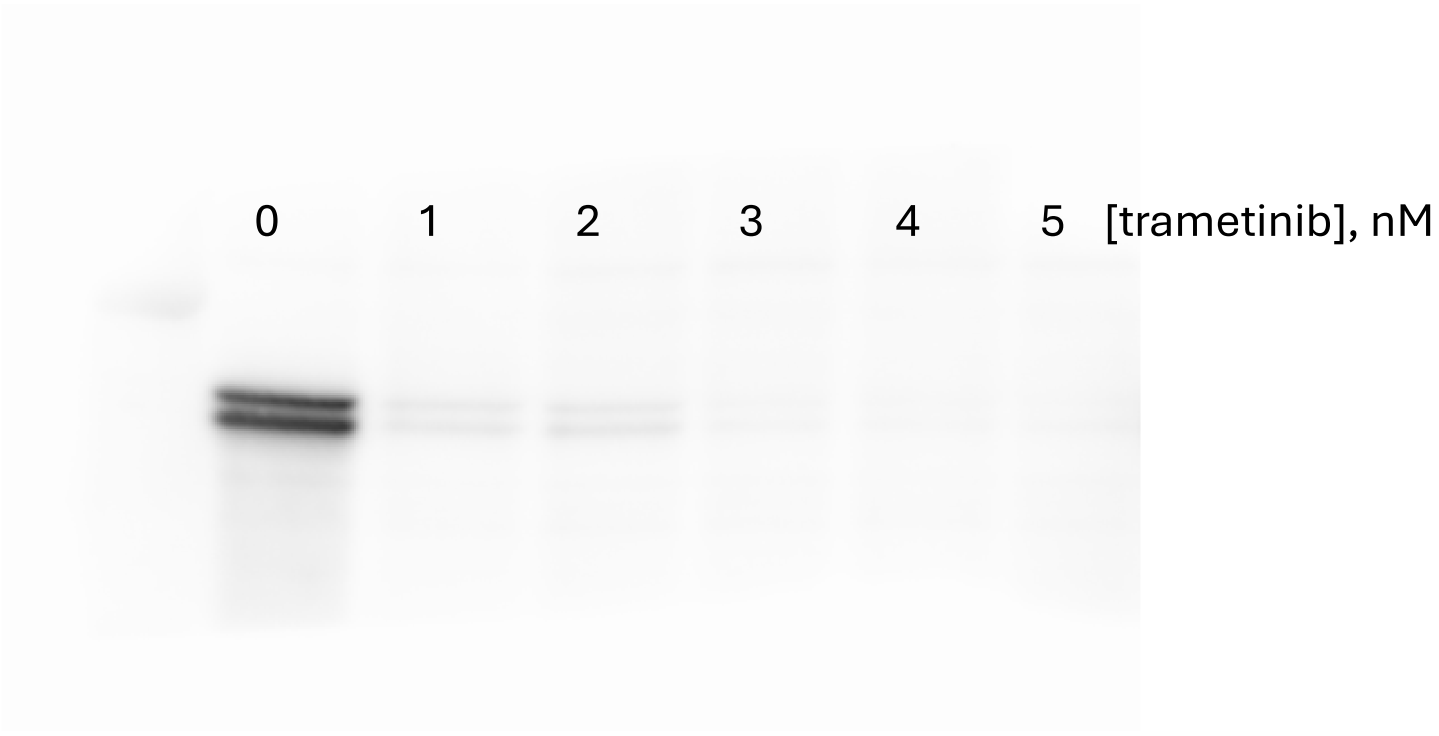

# Figure 2B: SK-N-BE(2)-C, vinculin

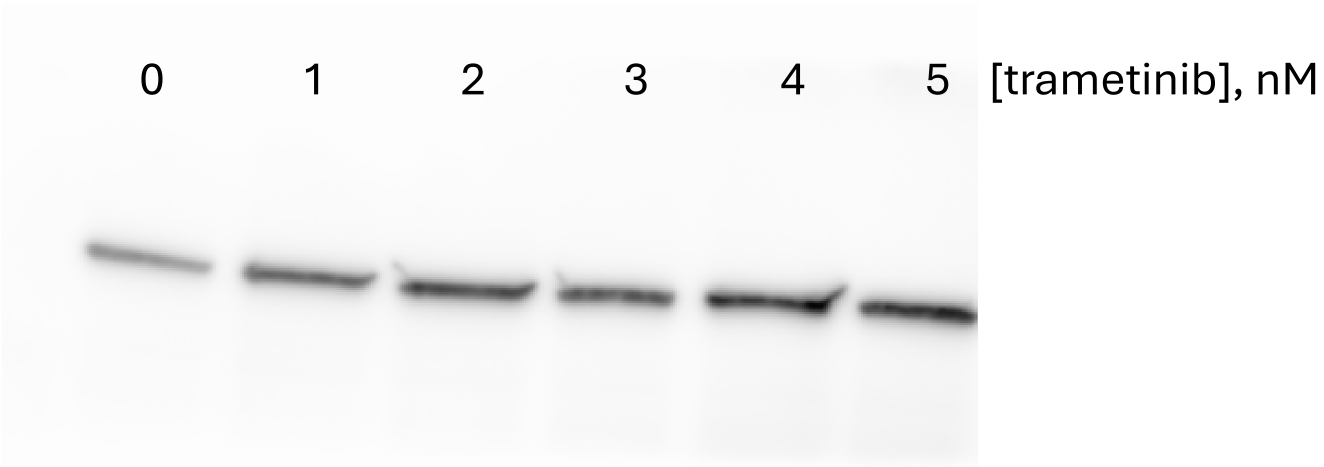

# Figure 3E: pERK

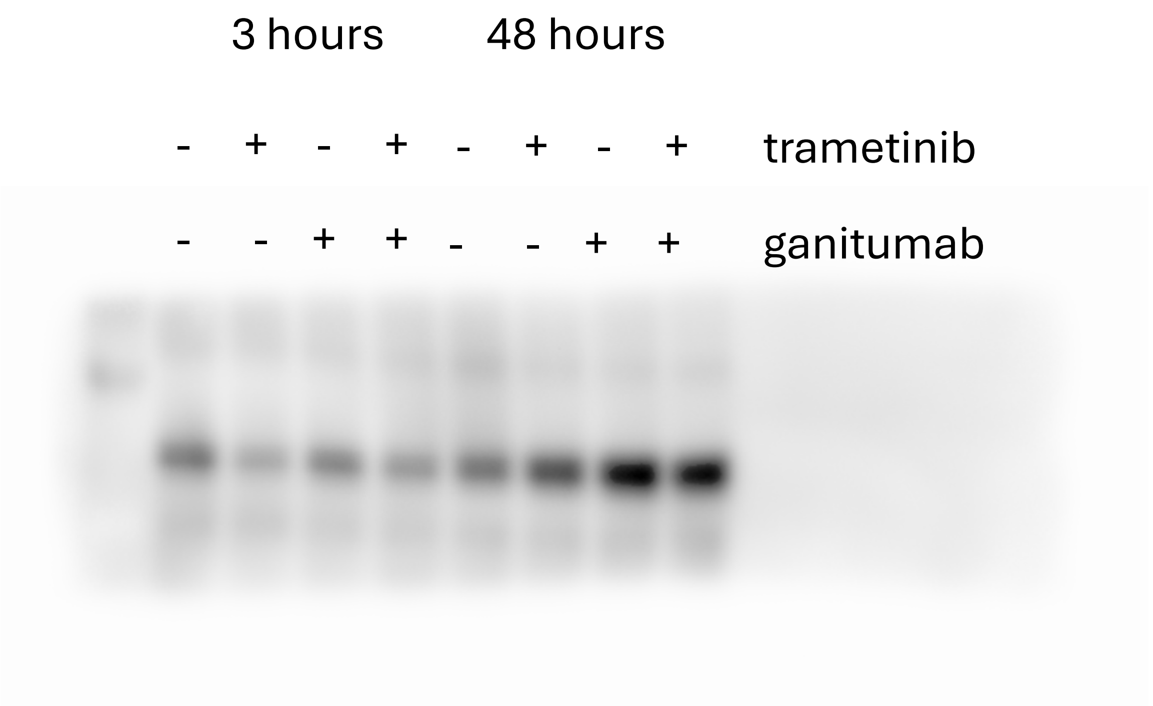

# Figure 3E: total ERK

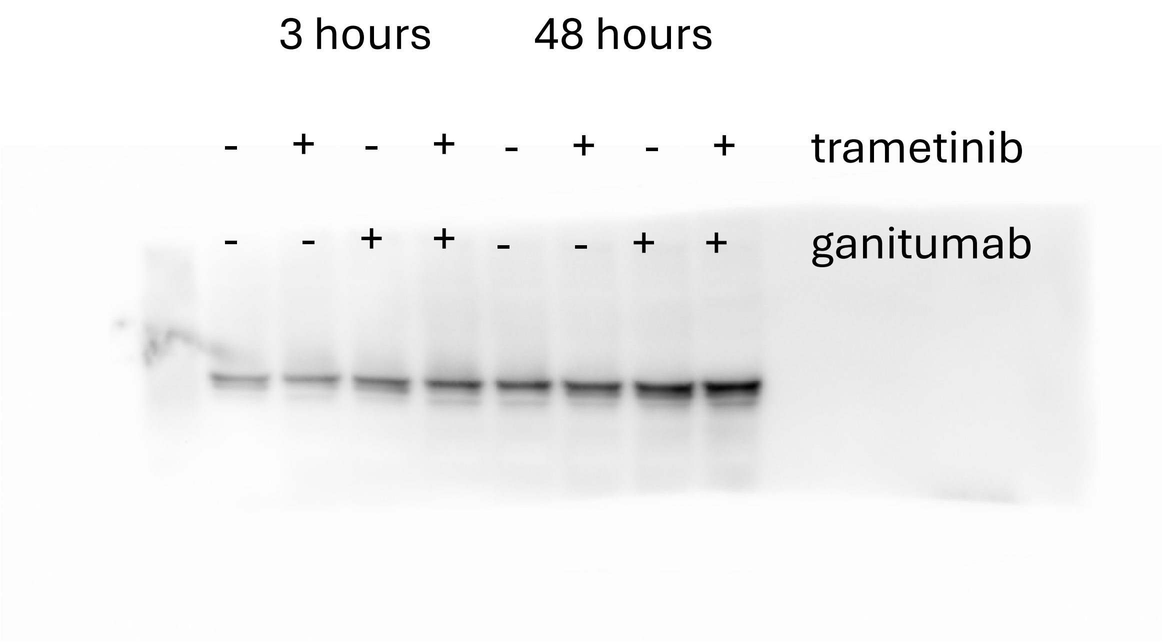

# Figure 3E: IGF1R

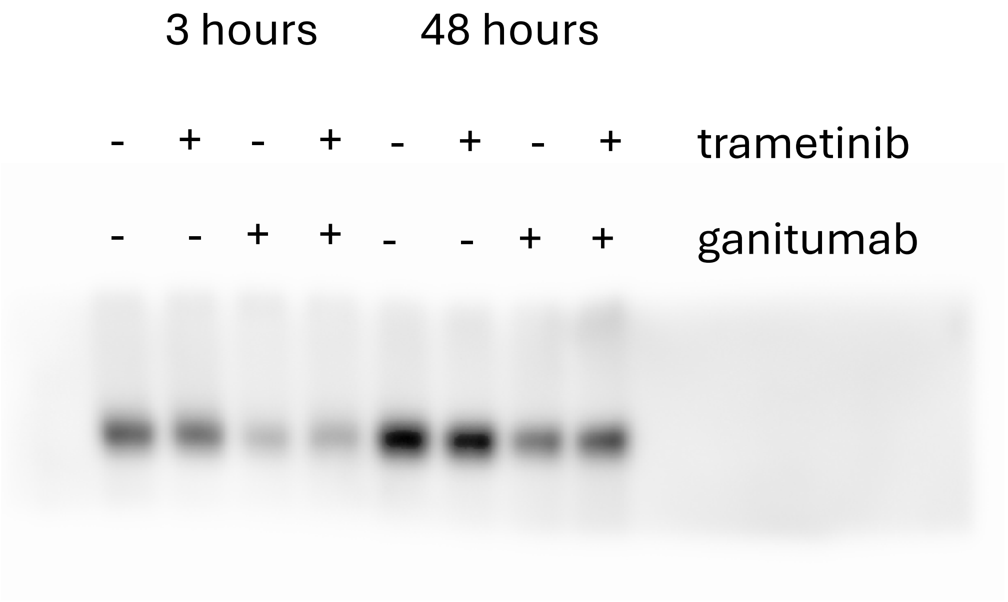

# Figure 3E: vinculin

3 hours 48 hours

- + - + - + - + trametinib

- - + + - - + + ganitumab

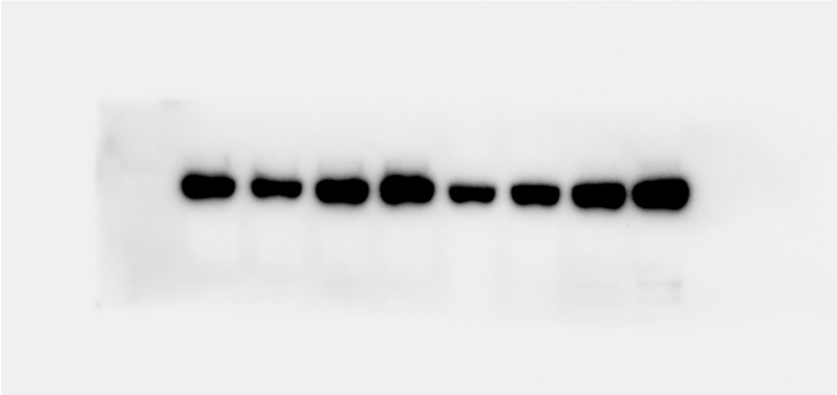

Supplement: Supplementary file 1 [file cancers-16-02320-s001.zip › cancers-3008576-supplementary-final/Supplementary file S1.pdf]
